# Collectanea

**Published:** 1828-08

**Authors:** 


### COLLECTANEA.

Floriferis ut apes in saltibus omnia libant,
Omnia nos, itidem, depascimur aurea dicta.

### PATHOLOGY.

*Cow-Pox produced from Grease in the Horse.*—Professor BERNDT has related the case, in HUFELAND's Journal, of a stable boy, who had neither been vaccinated nor had the small-pox. After dressing a horse affected with the disease known under the name of "Eaux aux Jambes," he had vaccine pustules in the hands. Several persons were inoculated with matter taken from these vesicles, and the true vaccine disease was produced. No effect was caused in those who had previously had either the vaccine or variolous disease.—*Bull. des Sciences Med.* *This is understood to be the true origin of vaccine*

*Anomalous Vision.*—A child, seven years of age, the son of a distinguished artist, had just began to receive instructions in drawing. To the astonishment of his father, he represented every object in an inverted position. If he drew a candle and candlestick, the former was placed downwards, the latter in the air. It was the same with chairs and tables, the legs were always placed upwards. The child was suspected of obstinacy and bad temper, and punishment was inflicted. He declared, however, that he represented every object in the position in which he saw them; and, as in every other respect they were very accurately sketched, he was at length believed. If the object he was about to draw was turned before it was given to him, he then represented it in its natural position; from which it was evident that the sensations communicated to the eye corresponded with the inversion formed upon the retina. This state of vision continued upwards of a year. From the age of eight years, the child saw objects in their natural position.

Cases analogous, if not precisely similar to this, have been before observed. Thus, a man saw every object in an inverted position for a considerable time. Houses appeared to him to rest upon their roofs, men to walk upon their heads, &c. This aberration of sight was produced by derangement of the digestive organs, and ceased with the cause which gave rise to it. Dr. WOLLASTON, after violent exercise of body and mind, suddenly found that he could only see half the figures of persons whom he met, or of any other object presented to him. Dr. CRAWFURD relates the case of a woman, who was attacked with a slight paralytic affection of the left side, after which she could only see the right half of objects; and, although she recovered the use of her limbs, her vision remained defective.—*Journ. Univ. des Sc. Med.*

*Suffocation caused by a Leech in the Larynx.*—A soldier suddenly experienced the usual symptoms of suffocation. His face was red and swollen, froth issued from his mouth, his eyes were directed upwards, and his breathing was nearly suspended. For a short time these symptoms abated, but quickly returned. As there were no signs of apoplexy, and the respiration alone appeared affected, it was suspected that there must be some foreign body in the air-passages, and the operation of laryngotomy was resolved upon. The patient, however, suddenly expired in a paroxysm of suffocation. Upon opening the body, a leech was found in the larynx. It adhered so tenaciously to the part, that it was removed with difficulty. The glottis had been obstructed by the presence of the animal, and the admission of air was thus rendered impossible.—*Ibid.*

---

*Hydatids in the Female Breast, resembling a Scirrhus Tumor.*—A robust young female, twenty-five years of age, complained of pains in the left breast, which at first had been confined to one point, but had subsequently extended, and became intensely severe. A hard tumor, of a shining appearance, about the size of a hen's egg, was found in the part. It was divided into several lobes, and resembled a deep-seated scirrhus. The precise nature of it, however, could hardly be determined, from the immense size of the breast. Various means were ineffectually tried to discuss the swelling, and, as the sufferings of the patient were intolerable, an operation was determined upon. The mammary gland was perfectly healthy, but beneath the pectoral muscle a cavity was discovered, filled with round bodies as white as snow. They were found to be hydatids; three of which were about the size of a nut, and seven much smaller. Most of them escaped freely through the wound. They were of a spherical form, and covered with a shining solid membrane of a white colour. The parietes of the cavity in which they had been contained were smooth, and resembled a serous membrane. To promote adhesive inflammation, lint was introduced into the wound. For a long time an ichorous fluid was discharged, and stimulating injections of nitric acid and mercury were found necessary to produce adhesion of the parts. The patient was cured in about two months from the operation.—*Clinique des Hopitaux.*

---

*Curious Cerebral Affection in four Brothers and Sisters.*—A woman in Norway was delivered, in 1801, of a healthy male child. He had very good health from the time of his birth, until about the seventh year. At that period his sight failed him more and more; his intellectual faculties and sensibility equally diminished; and, in the course of the ninth year, he became nearly blind, and perfectly insensible to external impressions: the sense of hearing appeared to be preserved the longest. Between the ninth and fourteenth year, he was attacked with epileptic fits, which became gradually more and more violent. At the age of fifteen, he was subject to frequent agitations, and uttered dreadful cries. He remained in this state until the age of twenty-one, when he died.

His sister, born some years after him, presented the same phenomena precisely at the same age. She had no menstrual discharge, and died at the age of twenty.

A few years after the birth of the last, the mother was delivered of a male infant, which, when he had attained his seventeenth year, was attacked with the same disease. At the time this account was written, he was between sixteen and seventeen years old, and in the same state of suffering which occasioned the premature end of his brother and sister.

At length a fourth, a female infant, of four years old, was sent to its relations living in a different quarter, under the fear that the metallic mines which are at Roeras might have some influence in the production of these distressing symptoms. This child enjoyed very good health until the age of seven years; but at this fatal period, the same phenomena observed in the three former were manifest, and, it is to be feared, will have the same melancholy termination.—*Bulletin des Sc. Med.*

---

*Hæmoptysis.*—If we inquire in what manner hæmoptysis from a ruptured blood-vessel can be occasioned by muscular exertion, it will be replied, that these causes increasing violently the action of the heart, a volume of blood is in consequence determined into the vessels of the lungs with a force sufficient to produce in their coats a solution of continuity. That this is the doctrine of the books will be perceived by a reference to CULLEN, p. 734 to 774 inclusive, and to GOOD's Study of Medicine, vol. iii. p. 175. Hæmoptysis from the rupture of a vessel is certainly not impossible: but we agree with Dr. GREGORY,* and Dr. CONDIE,† that it is of rare occurrence. The belief that the hemorrhage depends upon the rupture of a blood-vessel, the size of which is not unusually estimated by the quantity of blood discharged, not unfrequently paralyses the efforts of the practitioner, by leading him to suppose that the case is hopeless. The subject is, therefore, worthy a moment's consideration.

It is now satisfactorily ascertained that the vessels of a part, when labouring under irritation, have the power of exhaling blood, in the same manner they do serum or other fluids, and probably to as great an extent. In this manner very copious discharges of blood are sometimes thrown from the stomach in cases of hæmatemesis, in which disease the rupture of any particular artery is as uncommon as in hemorrhagic discharges from the lungs. In hæmoptysis the hemorrhage generally proceeds from the mucous membrane of the trachea or bronchia, a situation at which rupture of a blood-vessel very rarely, if ever, takes place. The cases of hæmoptysis upon record, presumed to have arisen from rupture, are very equivocal; the presumed source of the blood not being established by the appearances met with on dissection. In many instances, indeed, in which a fatal result has occurred, in cases succeeding almost immediately to some violent muscular exertion, the most accurate post-mortem examinations have shown that no rupture of any vessels in the lungs had taken place.‡ I have opened, observes BICHAT, a great number of subjects who have died during hemorrhage from the lungs, and, on examination of the bronchial surfaces, could never discover the least trace of erosion or rupture of the vessels, notwithstanding I have taken care to wash carefully these surfaces, to allow them to macerate, and to examine them with the glass. M. ANDRAL, who has recently, in the course of his observations upon pulmonary tubercles, paid much attention to the morbid appearances in the lungs of those who have died during hæmoptysis, confirms the statement of Bichat. He was able in no instance to recognise any other source of the hemorrhage than the surface of the mucous membrane of the bronchiæ. Excepting in those cases in which the hemorrhage was complicated with tubercles, the bronchial membrane presented the same appearances as in simple bronchitis. In some instances it was pale, or at most presented but a slight-coloured rose tint.

MORGAGNI mentions the appearances he met with after death, in cases of

* Elements of the Practice of Physic, second edition, p. 312.

† Condie on Hæmoptysis, North American Medical and Surgical Journal.

‡ An interesting case of this kind is related by BAUMES, in his Dissertatio de Hæmoptoe. 1748.

hæmoptysis, to be engorgement of the lungs and tubercles, in the neighbourhood of which the vessels were dilated.

Portal relates the case of a young man that had laboured under hæmoptysis for many weeks, in whom the lungs, after death, were found to be perfectly sound, but the bronchial glands were engorged and covered with vessels greatly dilated, many of which terminated by open extremities in the cavity of the bronchia.

According to Laennec, two varieties only of hæmoptysis can arise from rupture of the blood-vessels. The first when an aneurism bursts into the trachea or bronchiæ, and the second when there is a rupture of a vessel into a tuberculous excavation. These two species of hemorrhage are, however, followed by almost immediate death, and can by no means explain the phenomena of a disease of so common occurrence as hæmoptysis.

Of hæmoptysis from ulceration of the lungs, we can say but little, morbid anatomy having presented us with but few direct facts upon this point. We may remark, that in those cases of hemorrhage from the lungs complicated with phthisis, which have been generally supposed to depend upon ulceration, the blood has been found, upon dissection, to have been effused from the mucous membrane of the bronchia, and not from the ulcerated portion of the lungs.

When we consider the causes and symptoms of hæmoptysis, the plan of treatment by which it is most effectually controlled, as well as the appearances discoverable after death, we are irresistibly compelled to view the disease as dependant upon an irritation of the mucous coat of the lower portion of the trachea, or of the bronchia, in consequence of which irritation its vessels are induced to take on a hemorrhagic action. By Laennec is described a species of violent and extensive hæmoptysis, or (as he terms it) pulmonary apoplexy, which he presumes to arise from an effusion of blood into the parenchyma, or the air-cells of the lungs.

Examinations after death present an exactly circumscribed induration of some portion of the lungs, of a very dark red colour. When cut into, the incised surfaces appear granulated; and, when scraped, there is detached from them a small portion of very dark half-coagulated blood.

Hæmoptysis is seldom dangerous from the mere discharge itself, but from its being connected with, or preceding, some organic lesion of the lungs.

### PRACTICAL MEDICINE.

*Case of Gout;* by M. Mestivier, Membre de l'Academie.—Gouty affections conceal themselves under so many different forms, that it is often very difficult for the most experienced physician to follow them through all their modifications. M. Mestivier has had the advantage of many years' practice in a country where gout is almost endemic, and the following case he adduces as a very curious one:

The Prince of Wagram, upwards of sixty years of age, of a bilious and sanguineous temperament, strong constitution, had for a long time been subject, as autumn came on, to attacks of gout, which increased in violence after each attack. The seat of the disease generally was in the feet. The year preceding the campaign of Moscow, the Prince had the severest attack he had ever had: it was necessary to have recourse to bleeding to reduce the inflammatory action: this had the effect, and the attack gradually declined, and went away in fifteen or twenty days.

In the campaign of 1812, the Prince, from the pressure of circumstances, was exposed to great fatigue, and obliged to use a great deal of exercise, and to this cause perhaps he owed an exemption from his usual attack. However, the privations he had suffered, and the influence of climate, visibly affected his health, and it was with some difficulty he reached Posen, where he was obliged to lay up in bed. M. Mestivier was called to see him, and found him suffering intensely: his face, and every part of the body, was of a deep yellow; his look sad and uneasy; lips dry and without colour; tongue a little moist, but covered with a thick yellow coating; great thirst; frequent hiccup on drinking; respiration short and oppressed; no cough or palpitations. The epigastric region, which the patient would not suffer to be touched, presented nothing particular, but within twenty-four hours it had become the seat of so acute a pain that the weight even of the shirt was insupportable. This pain, which the patient compared to a toothache, stretched over towards the right hypochondrium; the abdomen was soft to the feel, but sluggish in its action. For the last three days there had been no evacuation, although some gentle aperients had been used; the urine was scanty, red, and deposited a brick-coloured stuff, adhering to the vessel. Pulse was small, compressed, very quick, but regular.

Taking all the symptoms into account, the case was considered as one of gall-stones passing through the biliary canals. Ol. Ricini, and a smart cathartic enema, were prescribed. The oil was rejected, but from the other means there was so copious an alvine discharge that the patient fainted. No advantage accrued from this; the case became serious, the danger augmenting every hour.

It was now suspected that it might be a case of erratic gout, and warm irritating baths and sinapisms were ordered to the feet. He passed a sleepless night. The next day there was no amendment, and the patient was very low. A blister was applied to the episgastrium. Four hours after the application of the blister, the feet were examined: the left was reddened from the effects of the means used, but not in the least painful; the right, on the contrary, was much swollen, red, and excessively painful, and having a well characterised fit of gout. From this time the other and alarming symptoms gave way, and in a fortnight the Prince was completely recovered. When a man has once got the gout, he can't live without it.

---

*Critical Remarks on the Use of the Sulphate of Quinia, or Quinquinia, in the Treatment of Fevers;* by M. Vulpès, of Naples.—M. V. gives the preference to the sulphate of quinia in intermittents, suborbital neuralgia, dyspepsia, &c.; while the quinquinia in substance is preferable in the fevers considered by the ancients as putrid, and which are produced by sedative miasmata exhaled from individuals crowded in a small and ill-ventilated space. The author, a partisan of the Italian doctrine, distinguishes that fever called prison or hospital fever from contagious typhoid fevers, such as febris petechialis-flavar, which are by him considered as inflammatory fevers; and it is on this fact that he rests his opinion.

In the month of March, 1825, it happened that there was so great a crowd of patients at a Maison de Fous, that they were constrained to lodge them in a convent, which was unprepared for their reception. The filthiest among them were shut up in a confined dormitory, which was in a remarkably nasty state. Very speedily fever made its appearance, and which was first looked upon as petechial, and treated antiphlogistically. The disease made rapid progress, and became very fatal. The sulphate of quinia was then employed, which exasperated all the symptoms. The quinquinia in substance was next used, and with the happiest effects: it was administered to the amount of half an ounce daily.

The way in which M. Vulpès explains this is, by considering the quinquinia as a remedy not antifebrile, but antispasmodic. It has no effect on the fever, but it acts against that unknown state of the habit producing the periodical return of the paroxysms. It is besides tonic. When fever is the result of inflammation, the quinquinia will have no effect. When pain arises from the simple reaction of the habit against deleterious substances, which tend to weaken vitality in altering the humors, as in the case above cited, the quinquinia, then, in substance should be given, and the sulphate of quinia would not do instead of it: the quinquinia in substance acting as an antiseptic, the sulphate of quinia having no such properties. In fever from marsh miasmata, either form is good; the sulphate of quinia having, however, the preference, from the stomach being more likely to receive it without disgust.

M. Vulpès denies that these fevers ever depend on internal phlegmasiæ: it may chance that partial inflammations develop themselves during the course of these fevers, but that is only a symptom, and calls for local antiphlogistic treatment, as much as the general state of the patient calls for the use of tonics and the specific, the bark.

---

*Chlorine in Chronic Affections of the Lungs.*—A bleaching establishment having been removed into a situation notoriously damp, and where catarrhal affections were extremely common, M. Bourgeois was not a little surprised to observe that those employed in this establishment were less liable to these attacks than their neighbours. As chlorine is much used in such establishments, he attributed to it the preventive influence. It chanced that two people, one with chronic catarrh resembling phthisis, and the other with a vomica in the lungs, were perfectly cured after two or three months' residence in this bleachery.

This substance (chlorine) has been used medicinally by Laennec at La Charité, and he considered it as meriting further trial. In its administration, M. B. would prefer its being disengaged from a mixture of peroxide of manganese and by the chloric acid, and mixed with the atmospheric air, and not inhaled by itself.

This agent has also been tried by MM. Louer, Villermay, Husson, Chomel, and Kergaradec, and, although without any decided success, they consider it as likely to be sometimes an useful addition to our remedial measures in phthisis.

---

*Case of Chronic Headache, successfully treated with Fowler's Arsenical Solution.*—Dr. Otto relates the following case:

Y. K., at the age of eleven years, was attacked, without any assignable cause, with violent headache that came on daily. His constitution was good, and with this exception he had been unusually healthy through life. When I first saw him (March 1819) it had continued three years, and had been gradually progressing in violence, but he thought he suffered rather most in summer. There had been no respite to the complaint, except during a few days when he was taking emetics. The paroxysms did not return at the same hour, and could be brought on at any time by exposure to the sun, over exertion, putting on a tight hat, or by any thing that bound his head considerably, which was rather disproportionably large. Ultimately the attacks lasted several hours, but varied a good deal both as to violence and duration, and were never accompanied by nausea or fever, nor seemed to depend on any thing that had been eaten. The pain was not confined to a small part, as is very often the case in periodical headache arising from intermittent fever, nor, like hemicrania, did it occupy one side of the head: the forehead suffered most, and was generally warm, but no part was entirely exempt from pain. He had taken very little medicine; for a physician of eminence, in the part of the country from which the family had lately removed, gave an assurance that it would be to no purpose, and great reliance was placed on his opinion. The parents were unwilling to have recourse to any thing painful, or even disagreeable, without a prospect of benefit. During two years the child continued at school, but afterwards he was obliged to lay aside his studies.

His case was stated to me while in attendance on his brother. I represented to them the impropriety of abandoning him to the operation of nature, which had been of no service, and that it was time enough to despair of relief when the resources of medicine had been fairly tried, and failed. Upon their consenting to his becoming my patient, I directed five drops of Fowler's solution of arsenic to be given him three times a day. This treatment was continued two weeks without any improvement in his complaint; nor was any nausea, griping, or swelling of the eyelids or face, produced. A day or two afterwards, without his taking any other medicine, he ceased to be attacked; nor was there any subsequent return of the disease for two years, when the patient removed to a distant part of the country to live, and I have not heard from him since. He was directed to be particular to avoid all exciting causes, to keep his bowels open, and to use but little animal food.

As the good effects of medicine are not always immediate, I am induced to think that the arsenical solution ought to be considered as having been instrumental in relieving him.—*North American Med. and Surg. Journal.*

---

*Deafness cured by Electricity.*—A girl, eight years of age, lost the power of hearing and speaking, during the course of an acute disease under which she laboured. Electricity was applied both by shocks and sparks, which were directed as much as possible to the affected organs. So complete had been the deafness, that she could not hear a pistol discharged close to her head. Under the influence of the electricity, however, she soon recovered her hearing. She had not regained her speech when the case was reported.—RUST'S *Magazine.*

---

*Herpes cured by Diet and Mercurials.*—A child, four years of age, was covered from head to foot with a scaly eruption; his health was also much impaired. He suffered so much from a troublesome itching of the whole body that he was with difficulty restrained from injuring himself by scratching. All the ordinary methods of treatment having failed, it was determined to try the effects of abstinence, which has been so much extolled by the German physicians. The patient was consequently confined to a very severe regimen, without, however, being entirely deprived of food. Mercurial frictions, with the Neapolitan ointment, were also frequently used. He was quickly and completely cured. The success is attributed more to the strict diet enforced than to the frictions.—*Ibid.*

---

*Contraction of the Œsophagus relieved by the internal Administration of Sal Ammoniac.*—A man, who had previously enjoyed a good state of health, was attacked in the following manner: He could swallow without much inconvenience both solid and liquid food, but, in about ten seconds afterwards, the former returned from the stomach into the pharynx, and he was incapable of swallowing the mass a second time without much torture, which increased every day. This kind of rumination did not occur with liquids. The patient was pale, and of a melancholic countenance. Upon attentively watching him during the act of eating, it was perceived that the mass did not pass into the stomach upon the first deglutition, as he imagined, but that it remained for some time near the cardiac extremity of the œsophagus, and that his distress was caused by its subsequent passage into the stomach. If the portion of food was very small and well masticated, he suffered but little. He described the pain he felt as if there was a wound in the passage, which was irritated by the food that passed over it.

M. Pagenstecher conceived that it was a case of inflammatory contraction of the œsophagus, near the cardiac orifice. Leeches were applied, and a decoction of marshmallows and nitre was prescribed. Liquids only were taken. The patient was not relieved, and he was in danger of dying from hunger.

It was now determined to employ the sal ammoniac, which had been so strongly recommended by Fischer, of Dresden, in such cases. Two drachms of it were mixed in two ounces of syrup of marshmallows, and an equal quantity of confection of elder. Half a spoonful was taken every two hours. The patient was informed of the slightly corrosive nature of the remedy. On the next day he was much relieved. The medicine caused a trifling burning sensation. He could swallow fluids without any difficulty. His amendment was progressive, and in ten days he was entirely cured. The sal ammoniac was continued for some weeks, the dose of it being gradually diminished.

A second case is related, in which the same success was obtained from similar treatment.—Von Hufeland's *Journal.*

---

### SURGERY.

*Removal of the Tonsils.*—The frequent occurrence of the disease requiring the operation of removing the tonsils, should be deemed a sufficient apology for calling the attention of the profession to a subject apparently so simple in itself, and which is rendered still more so from the various improvements suggested by the surgeons of this country, as well as by those of Europe.

Of late years, the removal of the tonsils by the canula and silver wire was considered the only safe and practicable mode of removing those glands. The mode of operating was by means of a double canula, from three or four inches in length, armed with a silver wire; the latter to be placed round the tumor, and to be drawn with a degree of tightness requisite to strangle the part enclosed, and the wire to be tightened from time to time, until the part dropped off; which generally was accomplished within four or five days. To Dr. PHYSICK the credit is due of shortening the suffering of the patient, by removing the wire after the first twenty-four hours, leaving the slough to separate by the process of nature; and by substituting an iron wire instead of silver, which is more readily disengaged from its attachments: but even with these improvements the patients suffered much, not only from the pain of the application, but from the inflammation and fever which is attendant upon this mode of operating. All other modifications of this plan are very unimportant. Having had an opportunity, during my late residence in France, of frequently witnessing the successful removal of the tonsils by the knife, and having, since my return, performed that operation in six instances with the same favorable results, I avail myself of the opportunity afforded me, through the medium of this Journal, to call the attention of the profession to the manner of conducting this operation, and the advantages it possesses over every other mode of removing the diseased tonsils. Nothing more is necessary than to pass a hook through the body of the enlarged gland, and to raise it from its bed between the arches of the palate; then to pass a probe-pointed bistoury under its base, and with one stroke to sever it from its connexion. The hemorrhage never exceeds two ounces, and is always to be arrested by a draught of cold water.

The following case is illustrative of the advantages which this mode has over all others before mentioned:

A young lady, fourteen years old, suffered great inconvenience for a long time from an enlargement of both tonsils. Dr. BROWN, the attending physician, consulted me as to the practicability of removing the tonsil of the opposite side by means of the knife: to this there could be no objection. Having assured her that it would be attended with a momentary pain, and that very inconsiderable compared with what she had experienced, she consented to the operation. It was performed in the short space of half a minute. The patient instantly declared that the pain bore no comparison to her sufferings caused by the operation recommended by Dr. Physick; nor afterwards did she experience the least inconvenience; even the next day she was enabled to walk out for pleasure.

The objection usually advanced to this practice arises from an erroneous idea as to the vascularity of the tonsil, and the hemorrhage to be apprehended. Referring to the vessels of the throat and posterior nares, we find that the artery which nourishes the tonsil is so indirect in its origin as to have the force of the circulation expended before it reaches its destination, being a branch of the third branch of the facial, which again is the third branch of the external carotid, making in all no less than six branches. Under these circumstances, hemorrhage to any great extent is certainly not to be expected. The facts, too, on this subject are so numerous as to banish all apprehensions of danger from this source.—Dr. HOSACK *in the American Journal of Medical Sciences.*

